# Supplementary material for: Establishment of Novel Mouse Model of Dietary NASH Rapidly Progressing into Liver Cirrhosis and Tumors
Source: Cancers (Basel). 2023 Jul 24;15(14):3744. doi: 10.3390/cancers15143744 (PMC10378543; doi:10.3390/cancers15143744)

Supporting information

Index of supplementary figures and tables:

Supplementary Table S1. Composition of AIN93M and OYC-NASH2.

Supplementary Table S2. Primer pairs used for qPCR analysis.

Supplementary Figure S1. Experimental design and time course of weight change in C57BL/6J mice fed the OYC-NASH2 diet for 60 weeks.

Supplementary Table S1. Composition of AIN93M and OYC-NASH2.

	Name of nutrient	Unit	AIN-93M	OYC-NASH2
**General ingredients**	Water	g/100g(diet)	9.0	9.0
	Crude protein	g/100g(diet)	12.7	17.1
	Crude fat	g/100g(diet)	4.3	27.4
	Rough grey	g/100g(diet)	3.0	2.9
	Crude fiber	g/100g(diet)	5.0	4.7
	NFE	g/100g(diet)	65.9	39.0
	Calories	kcal/100g(diet)	353.5	470.5
**Calorie ratio**	Protein calorie ratio	%/ calorie	14.4	14.5
	Fat calorie ratio	%/ calorie	11.1	52.3
	NFE calorie ratio	%/ calorie	74.5	33.2
**Main fatty acid composition**	Saturated fatty acid	%/total fatty acids	15.9	41.7
	Monounsaturated fatty acid	%/total fatty acids	23.8	46.4
	Polyunsaturated fatty acids	%/total fatty acids	60.1	10.1
**Others important ingredient**	Amount of methionine added	g	0.34	0.11
	Amount of choline added	g	0.18	-

NFE: Nitrogen-Free Extract

Supplementary Table S2. Primer pairs used for qPCR analysis.

Gene	Accession #	Primer sequence (5'-3')	
18S rRNA	NR_003278	F	5'-CACGGACAGGATTGACAGATTG-3'
		R	5'-CAGACAAATCGCTCCACCAA-3'
Acta2	NM_007392	F	5'-ACTGGGACGACATGGAAAAG-3'
		R	5'-GTTCAGTGGTGCCTCTGTCA-3'
Afp	NM_007423	F	5'-AGTTTCCAGAACCTGCCGAG-3'
		R	5'-ACCTTGTCGTACTGAGCAGC-3'
Casp1	NM_009807	F	5'-TACCTGGCAGGAATTCTGGA-3’
		R	5'-AGTCCTGGAAATGTGCCATC-3’
Ccl2	NM_011333	F	5'-AGGTCCCTGTCATGCTTCTG-3'
		R	5'-GGGATCATCTTGCTGGTGAA-3'
Ccnd1	NM_007631	F	5'-CCAGAGGCGGATGAGAACAA-3'
		R	5'-ATGGAGGGTGGGTTGGAAA-3'
Cdk4	NM_009870	F	5'-CCTGAGGACATACCTGGACAAAG-3'
		R	5'-CCGCTTAGAAACTGACGCATT-3'
Cd68	NM_001291058	F	5'-GAGGTTGTGACGGTACCCAT-3'
		R	5'-ACATTGTATTCCACCGCCAT-3'
Clec1b	NM_019985.3	F	5'-CTGGGGATCATGTCGGTCAC-3'
		R	5'-TGGTATCTCCACTTCGTGGC-3'
Clec4f	NM_016751.3	F	5'-TCACTACTGTGGGCTTGCAG-3'
		R	5'-GACTTAGGCCCCAGTCCTTG-3'
Col1a1	NM_007742	F	5'-ACATGTTCAGCTTTGTGGACC-3'
		R	5'-TAGGCCATTGTGTATGCAGC-3'
Cxcr3	NM_009910.3	F	5'-AGCCATGTACCTTGAGGTTAG-3'
		R	5'-GTCAGAGAAGTCGCTCTCGT-3'
Cxcr6	NM_030712.4	F	5'-ACTGGGCTTCTCTTCTGATGCC-3'
		R	5'-CACTACCAGGTACACACAGGG-3'
Ddit3	NM_007837	F	5'-CAGCGACAGAGCCAGAATAA-3'
		R	5'-GACCAGGTTCTGCTTTCAGG-3'
Itgam	NM_008401	F	5'-ATTCGGTGATCCCTTGGATT-3'
		R	5'-GTTTGTTGAAGGCATTTCCC-3'

Itga2	NM_008396.3	F	5'-GCGGCTGCTAATGCTAGTTC-3'
		R	5'-CCAACCAGTAGCCAGTTGCC-3'
Klrb1c	NM_001159904.2	F	5'-GGGATGAGTGTCTTAGTGCGAG-3'
		R	5'-CCAGTCTTGTGGGCACTCTAAA-3'
Lgals3	NM_010705	F	5'-GTACAGCTAGCGGAGCGG-3'
		R	5'-CGGATATCCTTGAGGGTTTG-3'
Myc	NM_010849	F	5'-AGCCCCTAGTGCTGCATGA-3'
		R	5'-GTTTGCCTCTTCTCCACAGACA-3'
Nrf2	NM_007527	F	5'-GGACATGGAGCAAGTTTGGC-3'
		R	5'-GGCCTCAGCCCATCTTCTTC-3'
Ncf1	NM_010876	F	5'-GCCCAAAGATGGCAAGAATAAC-3'
		R	5'-TAGTCAGCAATGGCCCGATAG-3'
Pcna	NM_011045	F	5'-AGGAGGCGGTAACCATAGAGA-3'
		R	5'-GAGACAGTGGAGTGGCTTTTG-3'
p16	NM_001040654	F	5'-ATGGAGTCCGCTGCAGACAGAC-3'
		R	5'-ACGTTGCCCATCATCATCACCTGA-3'
Spp1	NM_009263	F	5'-CTCCTTGCGCCACAGAATG-3'
		R	5'-TTGGAAGAGTTTCTTGCTTAAAGTCA-3'
Sqstm1	NM_011018	F	5'-GTGGGACAGCCAGAGGAACAG-3'
		R	5'-TGAGGGGTCTAGAGAGCTTGG-3'
Tgf	NM_011577	F	5'-GGAGAGCCCTGGATACCAAC-3'
		R	5'-CAACCCAGGTCCTTCCTAAA-3'
Timd4	NM_178759.4	F	5'-GTCCGCCTTCACTACAGAATCA-3'
		R	5'-CTGCAAAGACTCACTTGTTGTT-3'
Tnf	NM_013693	F	5'-CCACCACGCTCTTCTGTCTAC-3'
		R	5'-AGGGTCTGGGCCATAGAACT-3'
Tnfsf14	NM_019418.4	F	5'-AGCACATCTTACAGGAGCCAAC-3'
		R	5'-AGTAACCGGGCTCCATGGTC-3'
Vsig4	NM_177789.5	F	5'-GCACTCCTCTTTGGAAGCAAC-3'
		R	5'-TCCCTGAACCAGCAATGGTC-3'

F, forward sequence; R, reverse sequence.

Acta2, actin a2 smooth muscle aorta

Afp, alpha fetoprotein

Casp1, caspase 1

Ccl2, chemokine (C-C motif) ligand 2

Ccnd1, cyclin D1

Cdk4, cyclin-dependent kinase 4

Cd68, CD68 antigen

Clec1b, C-type lectin domain family 1, member b

Clec4f, C-type lectin domain family 4, member f

Col1a1, collagen type I alpha 1 chain

Cxcr3, chemokine (C-X-C motif) receptor 3

Cxcr6, chemokine (C-X-C motif) receptor 6

Ddit3, DNA damage-inducible transcript 3

Itgam, integrin alpha M

Klrb1c, killer cell lectin-like receptor subfamily B member 1C

Lgals3, lectin, galactose binding, soluble 3

Myc, myelocytomatosis oncogene

Nrf2, Nuclear factor erythroid 2-related factor 2

Pcna, proliferating cell nuclear antigen

p16, cyclin dependent kinase inhibitor 2A

Ncf1, Neutrophil cytosol factor 1

Spp1, secreted phosphoprotein 1

Sqstm1, sequestosome 1

Tgf, transforming growth factor beta 1

Timd4, T cell immunoglobulin and mucin domain containing 4

Tnf, tumor necrosis factor alpha

Tnfsf14, tumor necrosis factor (ligand) superfamily, member 14

Vsig4, V-set and immunoglobulin domain containing 4

Supplementary Figure S1. Experimental design and time course of weight change in C57BL/6J mice fed OYC-NASH2 diet.

(**A**) C57BL/6J mice were divided into two groups, the control group (n=41) was fed a AIN93M diet and the OYC-NASH2 group (n=74, two mice died at 60 weeks) was fed the OYC-NASH2 diet for 60 weeks. (**B**) Body weight and some organ weight ratios of mice during the feeding period. Data are expressed as the means ± SEM. **P* < 0.05, ***P* < 0.01 and ****P* < 0.001 between AIN93M diet group and OYC-NASH2 diet group. Con, control; eWAT, epididymal white adipose tissue; W, week of the treatment.

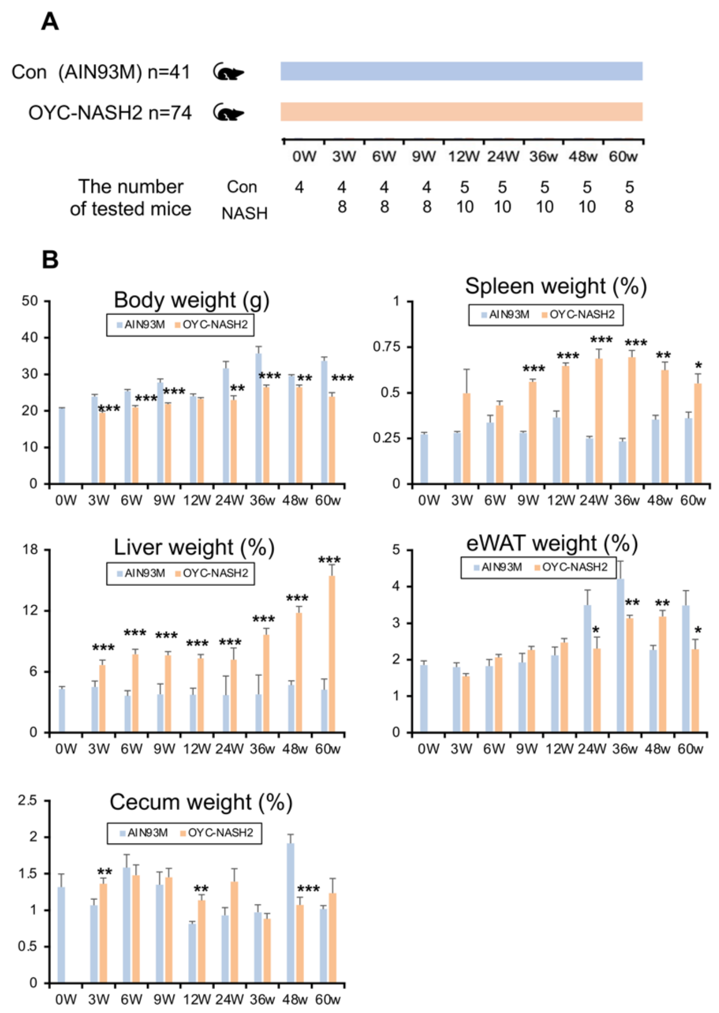

Supplement: Supplementary file 1 [file cancers-15-03744-s001.zip › cancers-2401662-supplementary.pdf]
